# The Secondary Metabolites and Biosynthetic Diversity From *Aspergillus ochraceus*

**DOI:** 10.3389/fchem.2022.938626

**Published:** 2022-08-25

**Authors:** Lin Chen, Erfeng Li, Wenqing Wu, Gang Wang, Jiaqian Zhang, Xu Guo, Fuguo Xing

**Affiliations:** ^1^ Comprehensive Utilization of Edible and Medicinal Plant Resources Engineering Technology Research Center, Zhengzhou Key Laboratory of Synthetic Biology of Natural Products, Zhengzhou Key Laboratory of Medicinal Resources Research, Huanghe Science and Technology College, Zhengzhou, China; ^2^ Horticulture and Landscape College, Tianjin Agricultural University, Tianjin, China; ^3^ Key Laboratory of Agro-Products Quality and Safety Control in Storage and Transport Process, Ministry of Agriculture and Rural Affairs, Institute of Food Science and Technology, Chinese Academy of Agricultural Sciences, Beijing, China

**Keywords:** *Aspergillus ochraceus*, secondary metabolite, structure, bioactivity, biosynthesis

## Abstract

*Aspergillus ochraceus*, generally known as a food spoilage fungus, is the representative species in *Aspergillus* section *Circumdati*. *A. ochraceus* strains are widely distributed in nature, and usually isolated from cereal, coffee, fruit, and beverage. Increasing cases suggest *A. ochraceus* acts as human and animal pathogens due to producing the mycotoxins. However, in terms of benefits to mankind, *A. ochraceus* is the potential source of industrial enzymes, and has excellent capability to produce diverse structural products, including polyketides, nonribosomal peptides, diketopiperazine alkaloids, benzodiazepine alkaloids, pyrazines, bis-indolyl benzenoids, nitrobenzoyl sesquiterpenoids, and steroids. This review outlines recent discovery, chemical structure, biosynthetic pathway, and bio-activity of the natural compounds from *A. ochraceus*.

## Introduction

Filamentous fungi in the genus *Aspergillus* are well known for their important roles in lifesaving drugs, devastating toxins, or mass-produced industrial enzymes. *Aspergillus* is currently subdivided into 27 sections by the physiologic, phenotypic, and DNA sequence data (Houbraken et al., 2020). *A. ochraceus*, the representative species in *Aspergillus* section *Circumdati* (Visagie et al., 2014), is generally known as a food spoilage fungus and is widely isolated from cereal, coffee, fruit, beverage, soil, and marine environments due to their environmental tolerance and fast growth. Ochratoxin A (OTA) makes *A. ochraceus* notorious for their role as contaminants in mycotoxigenic food and feed. Moreover, increasing cases have suggested *A. ochraceus* acts as human and animal pathogens causing onychomycosis (Xu et al., 2021), allergic bronchopulmonary aspergillosis (Hassanzad et al., 2019), and otomycosis (Ghibaudo and Peano, 2010). Some novel IgE-binding proteins have been identified from *A. ochraceus*, indicating the allergenic potency of mycelial proteins (Roy et al., 2021). Recently, *A. ochraceus* has been found in a SARS-CoV-2 positive immunocompetent patient in Iran (Koehler et al., 2020), and also can cause COVID-19 associated pulmonary aspergillosis (Hakamifard et al., 2021). On the other hand, the adaption of *Aspergillus* to shifting environments lead to the formation of a particular set of proteins (Day and Quinn, 2019; Wang et al., 2022b). *A. ochraceus* has been an important source of industrial enzymes like protease (El-Khonezy et al., 2021; Komarevtsev et al., 2021; Zhu et al., 2021), esterase (Romero-Borbón et al., 2018) and tannase (Aracri et al., 2019). Several medicinal metabolites, such as an intermediate for the synthesis of desogestrel and eplerenone, are characterized from *A. ochraceus* (Wang X. et al., 2020; Li et al., 2021). Some *A. ochraceus* strains have shown the mycoremediation potential to remove petroleum hydrocarbons (Bilen Ozyurek et al., 2021), and the remarkable capability for converting biodegradable waste to value-added end products for commercial applications (Jathanna and Rao, 2022).

**FIGURE 1:**
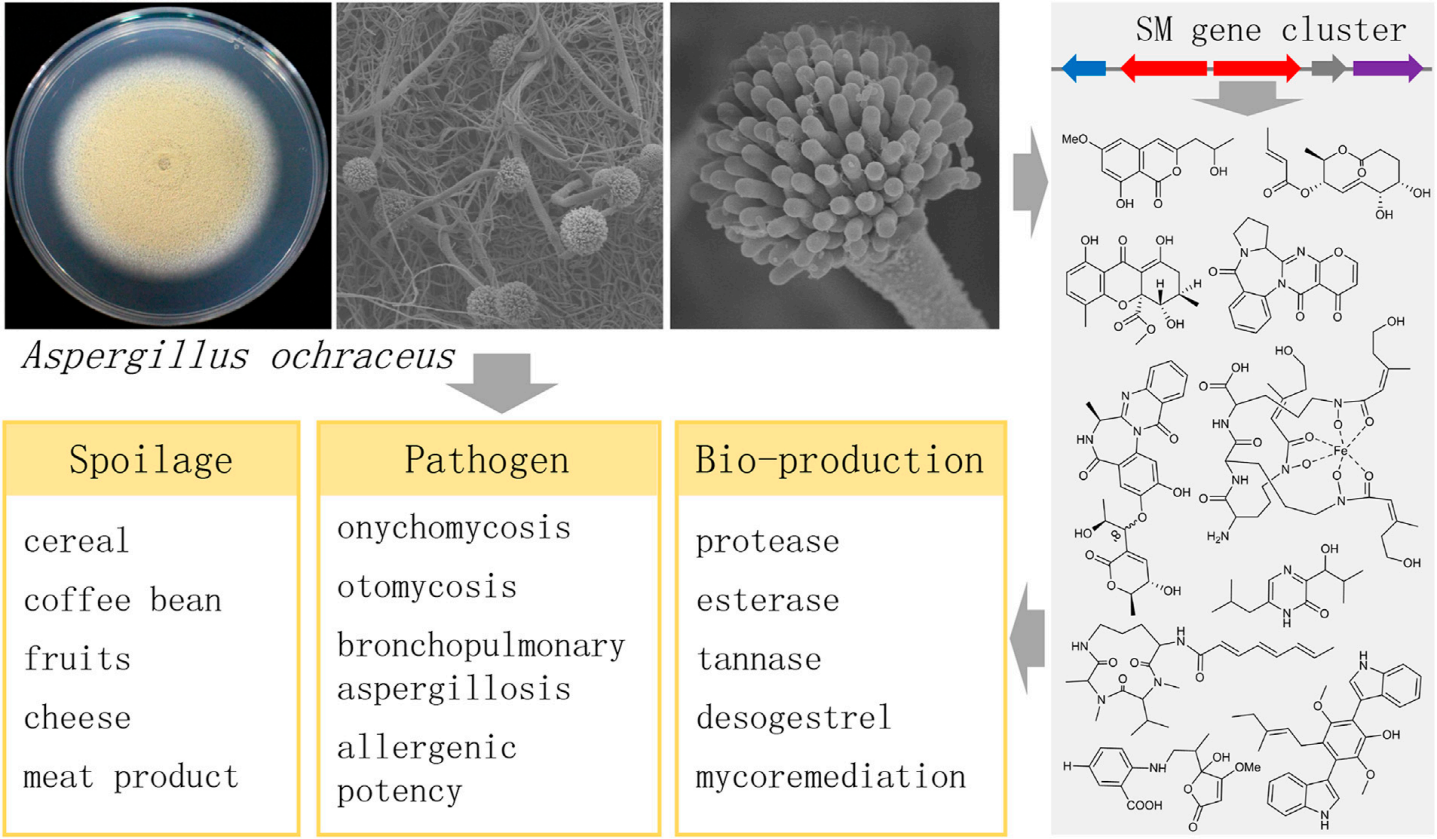
The important roles of the fungus *Aspergillus ochraceus*.

Secondary metabolites (SMs) play important roles both as a food spoilage fungus and as an industrial strain for bio-production (Figure 1). *A. ochraceus* as a food spoilage fungus exhibits a remarkably versatile secondary metabolism. Most SMs are derived from polyketides synthases, non-ribosomal peptides synthases, and terpene synthases, and used to defend their habitat or inhibit the growth of competitors (Macheleidt et al., 2016). And these compounds are likely to remain in the food chain after the occurrence of *A. ochraceus* in food substrate. From the perspective of drug discovery, many compounds have been isolated from *A. ochraceus* and screened for bio-activities. In fungi, the genes required for the biosynthesis of SMs are generally clustered on the chromosome. A growing number of *Aspergillus* genomes impressively shows numbers of unknown biosynthetic gene clusters (BGCs) of SMs, which considerably exceed the number of identified SMs, indicating their potential production of novel structural compounds (Keller, 2019). It has been well summarized that the linkage between SMs with their BGCs in the different *Aspergillus* spp. (Frisvad and Larsen, 2015; Romsdahl and Wang, 2019; Yu et al., 2021). However, although lots of compounds have been isolated from *A. ochraceus*, no other BGCs have been identified except for the OTA BGC. Reviewing the SMs and biosynthetic diversity from *A. ochraceus* would give insights into the understanding and utilization of this fungus.

In this review, we have critically scrutinized the existing reports to provide an overview of the various SMs produced by *A. ochraceus*. Considering the structural characteristics and biogenetics, these compounds could be classified as polyketides, nonribosomal peptides, diketopiperazine alkaloids, benzodiazepine alkaloids, pyrazines, bis-indolyl benzenoids, nitrobenzoyl sesquiterpenoids, steroids, et al. In addition, we present the bioactivities and the possible biosynthetic pathway of some compounds, which are ignored due to the shading of mycotoxin OTA.

## Secondary Metabolites From *A. ochraceus*

### Ochratoxins

**FIGURE 2:**
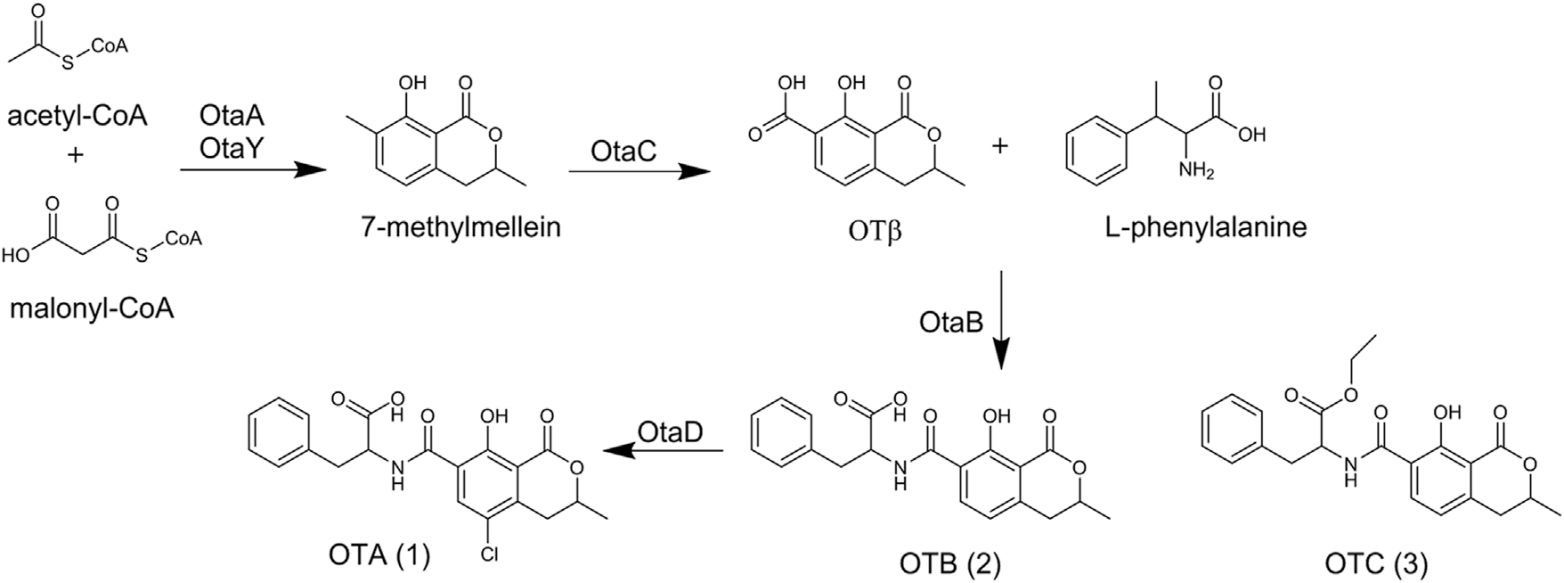
The structure and biosynthesis of ochratoxins.

The most common *A. ochraceus* product described is ochratoxin A (**1**) (Figure 2). It was first discovered from *A. ochraceus* isolated from sorghum grain in South Africa in 1965 (van der Merwe et al., 1965b). Since then, more than 90 kinds of foodstuffs such as cereal, beer, coffee, cheese, and meat products have been found to contribute to OTA dietary exposure (Ostry et al., 2013). Recently, some *A. ochraceus* strains were re-classified as *A. westerdijkiae* based on the *β-tubulin* sequence and morphological identification (Cui et al.; Durand et al., 2019). Surprisingly, a genome mining study demonstrated the integral cluster of OTA was not found in the *A. ochraceus* genome, (Gil-Serna et al., 2020), while the strain *A. ochraceus* fc-1, re-classified as *A. westerdijkiae*, has been reported to contain an intact cluster (Wang et al., 2018; Wang et al., 2022a).

Biological toxic studies performed on rats, trout, and mice demonstrated the carcinogenic potency of OTA (Kanisawa and Suzuki, 1978). The International Agency for Research on Cancer evaluated the experimental evidence for carcinogenicity as sufficient and classified OTA as a possible human carcinogen (group 2B) (IARC, 1993). Additionally, OTA was well documented in its nephrotoxicity, immunotoxicity, myelotoxicity, genotoxicity, embryotoxic, and teratogenicity in many species (Pfohl-Leszkowicz and Manderville, 2007; Malir et al., 2013a; Malir et al., 2013b).

OTA consists of a para-chlorophenolic moiety containing a dihydroiso-coumarin group that is amide-linked to l-phenylalanine. OTB (**2**) and OTC (**3**) were also isolated from *A. ochraceus* as the dechloro and ethyl ester derivatives of OTA. In similar toxicity tests, (**2**) and (**3**) were proved to be non-toxic at a thousand-fold higher dose level compared with (**1**) (Van der Merwe et al., 1965a). The metabolism of OTA has been extensively studied over the past decades. After *in vitro* incubation of OTA with the microsomes of human, rat, and pig, hydroxylated derivatives 4(R)-OH-OTA, 4(S)-OH-OTA (Størmer et al., 1981) and 10-OH-OTA (Størmer et al., 1983) have been detected. The α-chymotrypsine and carboxypeptidase from homogenates of the pancreas and small intestine led to the cleavage of the peptide bond in OTA and yield OTα (Hansen et al., 1982). Several derivatives occur naturally in the animal body by biotransformation, including OTA open lactone (OP-OA) (Gillman et al., 1999), OTA quinone (OTQ) (Gillman et al., 1999), OTA hydroquinone decarboxylated (DC-OTHQ) (Faucet-Marquis et al., 2006), conjugate OTA quinone-glutathion (OTQ-Glutathion) (Dai et al., 2002), OTA methyl ester (OTA-Me) (Li et al., 2000), Ethylamide OTA (OE-OA) (Xiao et al., 1995), tyrosine OTA (OTA-tyrosine) (Creppy et al., 1990) and so on (Malir et al., 2016).

The biosynthetic pathway of (**1**) was first investigated by exploring the related metabolites. Labeling study by the introduction of [1-^14^C] *l* -phenylalanine into the culture of *A. ochraceus* lead to the detection of radioactivity in the phenylalanine moiety of (**1**), indicating *l* -Phenylalanine was the precursor of (**1**) (Steyn et al., 1970). [2-^14^C]acetate and [2-^14^C]malonic acid radiolabeling experiments indicated malonic acid was involved in the isocoumarin moiety biosynthesis but not in the phenylalanine moiety biosynthesis (Ruhland et al., 1996), and the isocoumarin moiety most derived *via* acetate condensation.

Advances in sequencing technology make scientists realize the dihydrocoumarin moiety of (**1**) is catalyzed by polyketide synthase (PKS) and the polyketide moiety of (**1**) is linked to *l* -phenylalanine catalyzed by non-ribosomal peptide synthetase (NRPS) (Huffman et al., 2010; Wang et al., 2015). In 2018, a consensus biosynthetic gene cluster was identified by comparative genomic analyses among OTA-producing fungi. And the biosynthetic pathway was clarified by discovering the intermediate metabolites in OTA gene disruption mutants. Briefly, OtaA (PKS) utilized acetyl-CoA and malonyl-CoA to synthesize 7-methylmellein by condensation, which was oxidized to OTβ by cytochrome P450 OtaC. Then, OtaB (NRPS) combined OTβ and *l* -phenylalanine to synthesize (**2**) by catalyzing the formation of an amide-bond. (**2**) was chlorinated by the halogenase OtaD to form (**1**). Recently, a cyclase gene *otaY* was proved to be involved in the biosynthesis of (**1**) (Figure 2). OtaY was speculated to catalyze the cyclization process of 7-methylmellein (Ferrara et al., 2020; Ferrara et al., 2021).

### Polyketides

Polyketides occur in various organisms including fungi, bacteria, and plants. They are recognized as one of the most important categories of SMs. Polyketides have a common biosynthetic origin of small carboxylic acids such as acetate, propionate, and, rarely, butyrate, with diversity in structure (Palmer and Alper, 2019). Several polyketides have been isolated from *A. ochraceus*.

**FIGURE 3:**
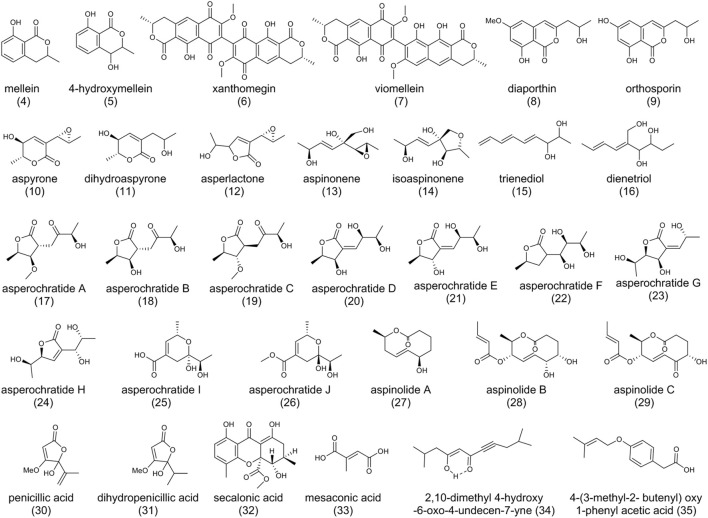
The structure of polyketides in *Aspergillus ochraceus*.

As shown in Figure 3, mellein (**4**) and 4-hydroxymellein (**5**), structurally similar to the dihydroisocoumarin moiety of (**1**), are also produced by *A. ochraceus* (Cole et al., 1971; Moore et al., 1972). The biosynthesis of (**4**) starts with the condensation of acetyl-CoA and malonyl-CoA, and acetyl units would be added until the pentaketide is formed (Huff and Hamilton, 1979), just like the biosynthesis 7-methylmellein in OTA. Compound (**4**) and its derivatives exhibit an array of bio-activities such as antitumor, antifungal, antibacterial, and anti-inflammatory (Hussain et al., 2015; Mdachi, 2016).

Xanthomegnin (**6**) and viomellein (**7**) are mycotoxins produced by *Penicillium viridicatum*, *A. melleus*, *A. sulphureus*, as well as *A. ochraceus* (Stack and Mislivec, 1978; Kamiya et al., 2017). It was reported that the toxicity of *P. viridicatum* strain 66-68-2 was due to (**6**), (**7**), rubrosulphin, viopurpurin, and brevianamide A, instead of (**1**) or citrinin (Stack et al., 1977). Gene inactivation experiments suggested (**6**) and (**7**) originated from the same polyketide pathway (Nicolaisen et al., 1996; Kandemir et al., 2015).

Diaporthin (**8**) and orthosporin (**9**) were also characterized from *A. ochraceus* (Harris and Mantle, 2001). (**8**) was reported to reproduce symptoms of canker in Chestnut trees and (**9**) could cause irregular brown spots on the leaves of oats (Hallock et al., 1988). The difference between the two compounds is the replacement of the methoxy by hydroxyl moiety on the benzene ring. Structurally, we hypothesize that one PKS could be responsible for the biosynthesis of these compounds.

Another array of SMs found in *A. ochraceus* was aspyrone (**10**), dihydroaspyrone (**11**), asperlactone (**12**), aspinonene (**13**), isoaspinonene (**14**), trienediol (**15**), and dienetriol (**16**), with the bioactivities of anti-microbial and anti-nematode (Fuchser et al., 1994; Kimura et al., 1996; Fuchser and Zeeck, 1997; Yurchenko et al., 2019). (**10**) and (**13**) have structural similarities and belong to the growing family of fungal epoxides. They contain a C_9_ carbon skeleton and one oxirane ring at a similar position and share the same biosynthetic pathway. The unknown PKS catalyzes the biosynthesis of an intermediate metabolite β-hydroxy acid, followed by modification of post-polyketide enzymes. A rearrangement of the carbon skeleton forms a branched pentaketide, and the aldehyde intermediate is either oxidized or reduced to yield (**10**) and (**13**), respectively (Fuchser et al., 1995; Fuchser and Zeeck, 1997). Recently, asperochratides A-J (**17**-**26**), which belong to aspyrone co-metabolites, have been isolated from *A. ochraceus* and found to exert significant cytotoxic effects on BV-2 cell line (Zou et al., 2020). Aspinolides A-C (**27**-**29)** are pentaketides with different precursors from (**10**)/(**13**), indicating the different PKS pathways. Generally, PKS catalyzes the biosynthesis of an intermediate metabolite hydroxy acid, followed by cyclization by a thioesterase to form a 10-membered lactone. The intermediate lactone is further modified following two pathways (reductase and acylase) to form (**28**) and (**29**) (Fuchser and Zeeck, 1997). Given their interesting structure and bioactivity, total synthesis by different approaches has been explored (Pilli et al., 2000; Ghosh and Rao, 2007; Chowdhury et al., 2009).

Organic acids derived from PKS pathways such as penicillic acid (**30**) (Frank et al., 2019), dihydropenicillic acid (**31**), secalonic acid (**32**) (Yamazaki et al., 1971), and mesaconic acid (**33**) (Zou et al., 2017) were isolated from *A. ochraceus* stains. (**30**) caused significant problems in animal and human health, and (**32**) showed antimicrobial activity against *Bacillus subtilis* and *Piricularia oryzae*. 2, 10-dimethyl 4-hydroxy-6-oxo-4-undecen-7-yne (**34**) and 4-(3-methyl-2-butenyl) oxy 1-phenyl acetic acid (**35**) were actively produced in an *A. ochraceus* mutant strain by UV irradiation (Awad et al., 2005).

### Nonribosomal Peptides

Non-ribosomal peptides are synthesized by multi-modular NRPSs from building blocks of 20 kinds of proteinogenic amino acids and non-proteinogenic amino acids such as ornithine and β-alanine (Wang et al., 2016; Stevenson et al., 2019). They have been optimized for a certain function in the native producer during years of evolution, as well as represent a promising basis for the development of substances with excellent activities. Generally, most of the non-ribosomal peptides described from *A. ochraceus* are cyclic peptides.

**FIGURE 4:**
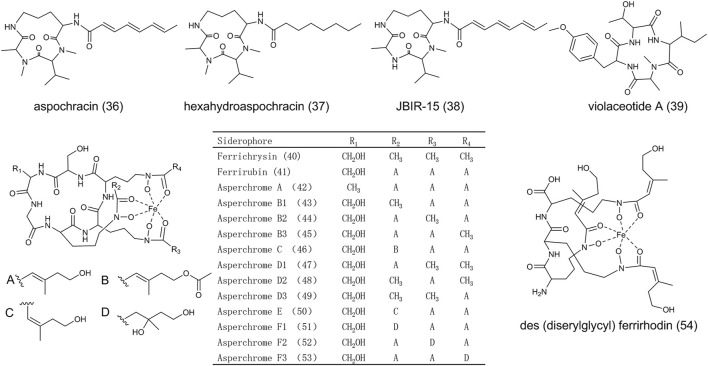
The structure of nonribosomal peptides in *Aspergillus ochraceus*.

As shown in Figure 4, aspochracin (**36**) is a cyclotripeptide composed of *N*-methy- *l* -alanine, *N*-methy- *l* -valine and *l* -ornithine (Chang et al., 1969). (**36**) demonstrated contact toxicity to the first instar larvae and eggs of silkworm. However, the insecticidal activity completely diminished in hexahydroaspochracin (**37**), indicating the triene in the side chain has been involved in molecular bioactivity. JBIR-15 (**38**), of which the N-methyl alanine is replaced by alanine compared to (**36**), was isolated from *A. sclerotiorum* (Motohashi et al., 2009). Violaceotide A (**39**) was extracted from a solid rice medium of *A. ochraceus* (Frank et al., 2019) and its structure was elucidated as cyclic tetrapeptide with l-threonine, L-O-methy-tyrosine, N-methy-l-alanine and l-lsoleucine. (**39**) showed anti-inflammatory activity with a high inhibitory rate (Liu et al., 2018).

Most fungi can produce siderophores under iron deficiency or other iron-related conditions. Hydroxamate-type siderophores, classified into fusarinines, coprogens, or ferrichromes, were mostly found and characterized in fungi (Garnerin et al., 2017). A large number of ferrichromes were isolated from iron-deficient cultures of *A. ochraceus* (Jalal et al., 1984). Ferrichromes are cyclic hexapeptides composed of three *N* ^δ^-acyl-*N* ^δ^-hydroxy- *l* -ornithine, one glycine, and two variable amino acids (alanine, serine, or glycine) linked by peptide bonds. The structures of ferrichrysin (**40**), ferrirubin (**41**), and asperchromes (**42**-**53**) were shown in Figure 4. Serine and alanine participate in the formation of these molecules as the variable amino acids; the acetyl and anhydromevalonyl are acyl groups linked to ornithine. Des (diserylglycyl)ferrirhodin (**54**) does not follow the typical ferrichrome structure. It is a linear siderophore consisting of three *N* ^δ^-cis-anhydromevalonic acid-*N* ^δ^-hydroxy- *l* -ornithine moieties linked by peptide bonds. The absence of a cyclic hexapeptide ring in des (diserylglycyl)ferrirhodin leads to a bathochromic shift withpH value decreasing from 2.0 to 1.7, indicating the change of iron-binding property (Jalal et al., 1984). It is rare among microorganisms that *A. ochraceus* produces various siderophores derived from a common cyclic hexapeptide ring with the *N*-acyl side chain surrounding the iron atom. Their diversity of function and structure is worthy to be further explored.

### Diketopiperazine Alkaloids

**FIGURE 5:**
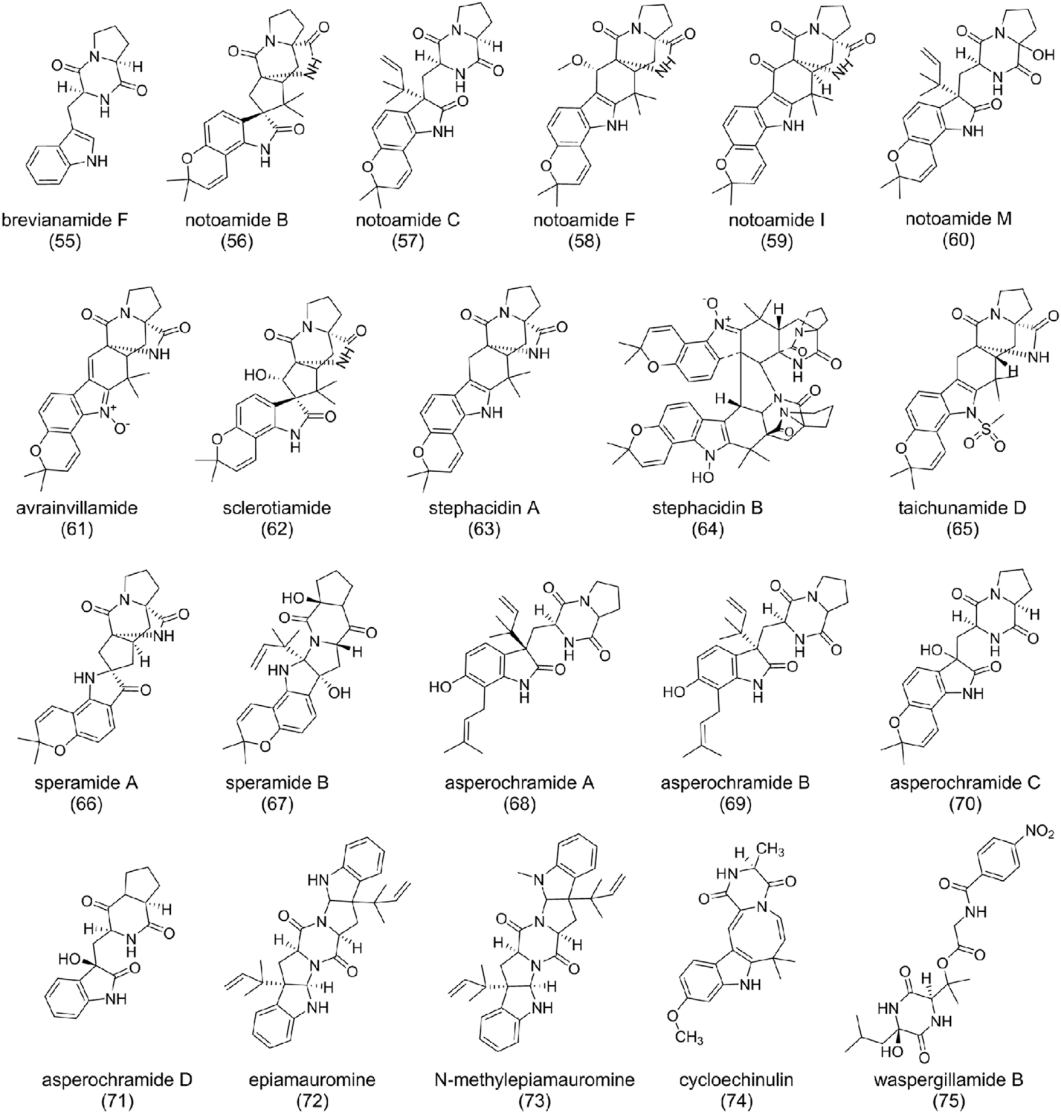
The structure of diketopiperazine alkaloids in *Aspergillus ochraceus*.

Diketopiperazine alkaloids are commonly isolated from fungi with excellent biological activities such as anticancer, antimicrobial, antiviral, antioxidant, and immunomodulatory (Hu et al., 2019; Wang M.-H. et al., 2020). Diketopiperazine alkaloids, with a stable six-membered ring backbone, are cyclic dipeptides formed by the condensation of two amino acids through peptide bonds by NRPS (Jia et al., 2019). *A. ochraceus* is capable of producing abundant diketopiperazine alkaloids with structural diversity and biological activity (Figure 5).

Indole diketopiperazine alkaloids are the main compounds in *A. ochraceus*, which were characterized by condensation of a complete tryptophan and other amino acids such as tryptophan, proline, and alanine (Ma et al., 2016). Brevianamide F (**55**), formed by condensation of a tryptophan and a proline without any modification, is the common precursor of many indole diketopiperazines. It was first isolated and characterized from *P. piscarium* and subsequently found in *A. ochraceus* (Vinokurova et al., 2003; Liu et al., 2018). Notoamide family compounds [B (**56**), C (**57**), F (**58**), I (**59**), and M (**60**)] were also isolated from *A. ochraceus* (Liu et al., 2018; Hu et al., 2021). (**56**) possesses the pyranoindole ring, with molecular similarity with avrainvillamide (**61**), sclerotiamide (**62**), and stephacidin A (**63**) (Sugie et al., 2001; Qian-Cutrone et al., 2002; Cui et al., 2009). Possible biosynthetic rules have been suggested: deoxybrevianamide E was first catalyzed to (**63**) then to (**56**), followed by branching to notoamide A or (**62**) (Kato et al., 2007). (**57**) and M (**60**) are prenylated indole diketopiperazine alkaloids, which contain diketopiperazine and isoprenoid moieties or structures derived thereof. Isopentenylation usually gives compound biological and pharmacological properties distinct from their non-prenylated precursors (Li, 2010). Brevianamide B (**56**) and C (**57**) showed moderate cytotoxicity against HeLa and L1210. It is worth mentioning that (**57**) can induce G2/M-cell cycle arrest at a concentration of 6.3 mg/ml (Kato et al., 2007). (**63**) showed cytotoxic activity against various human tumor cell lines, while bisindole diketopiperazine alkaloid stephacidin B (**64**), the dimer of (**63**), exhibited more potent antitumor activities (Qian-Cutrone et al., 2002). Taichunamide D (**65**), as an *N*-methylsulfonyl derivative of 6-epi-stephacidin A first isolated from *A. taichungensis*, was also found from *A. ochraceus* (Liu et al., 2018).

Speramides A (**66**) and B (**67**), featured by the fusion of a pyrrolidine ring to bicyclo-[2,2,2] diazaoctane subunit, were derived from the precursor (**55**). Since the structure of (**66**) has a similarity to (**63**), it is proposed that (**63**) could be converted to (**66**) through oxidation and rearrangement. Evaluation of their bioactivity demonstrated that (**66**) had moderate antimicrobial activities against *Pseudomonas aeruginosa* (Williams et al., 1990; Chang et al., 2016).

Asperochramides A-D (**68**-**71**) is another group of indole diketopiperazine alkaloids isolated from *A. ochraceus* (Liu et al., 2018). (**68**) and (**69**) are a pair of epimers assigned with the same planar structure. The significant difference between (**68**) and (**70**) is the cyclization of 2-isopentenyl and the replacement of 1-isopentenyl by hydroxyl. Removing the two isopentenyl forms (**71**). Bio-activities studies demonstrated that (**68**) has anti-inflammatory potential (Liu et al., 2018).

Most of the diketopiperazine alkaloids isolated from *A. ochraceus* are directives of precursor condensing of tryptophan and a proline. However, epiamauromine (**72**), which was stereochemically different from amauromine (Takase et al., 1985) characterized by condensation of two prenylated tryptophan, and N-methylepiamauromine (**73**) were isolated from *A. ochraceus* (de Guzman et al., 1992). Cycloechinulin (**74**) is formed by condensation of tryptophan and an alanine (de Guzman et al., 1992). And tetrapeptide diketopiperazine waspergillamide B (**75**) is a conjugate of p-aminobenzoic acid, Gly, hydroxy-Val, and hydroxy-Leu residues. Nitro-substituted diketopiperazines are rare compounds with excellent activity (Quezada et al., 2017; Frank et al., 2019).

### Circumdatins

**FIGURE 6:**
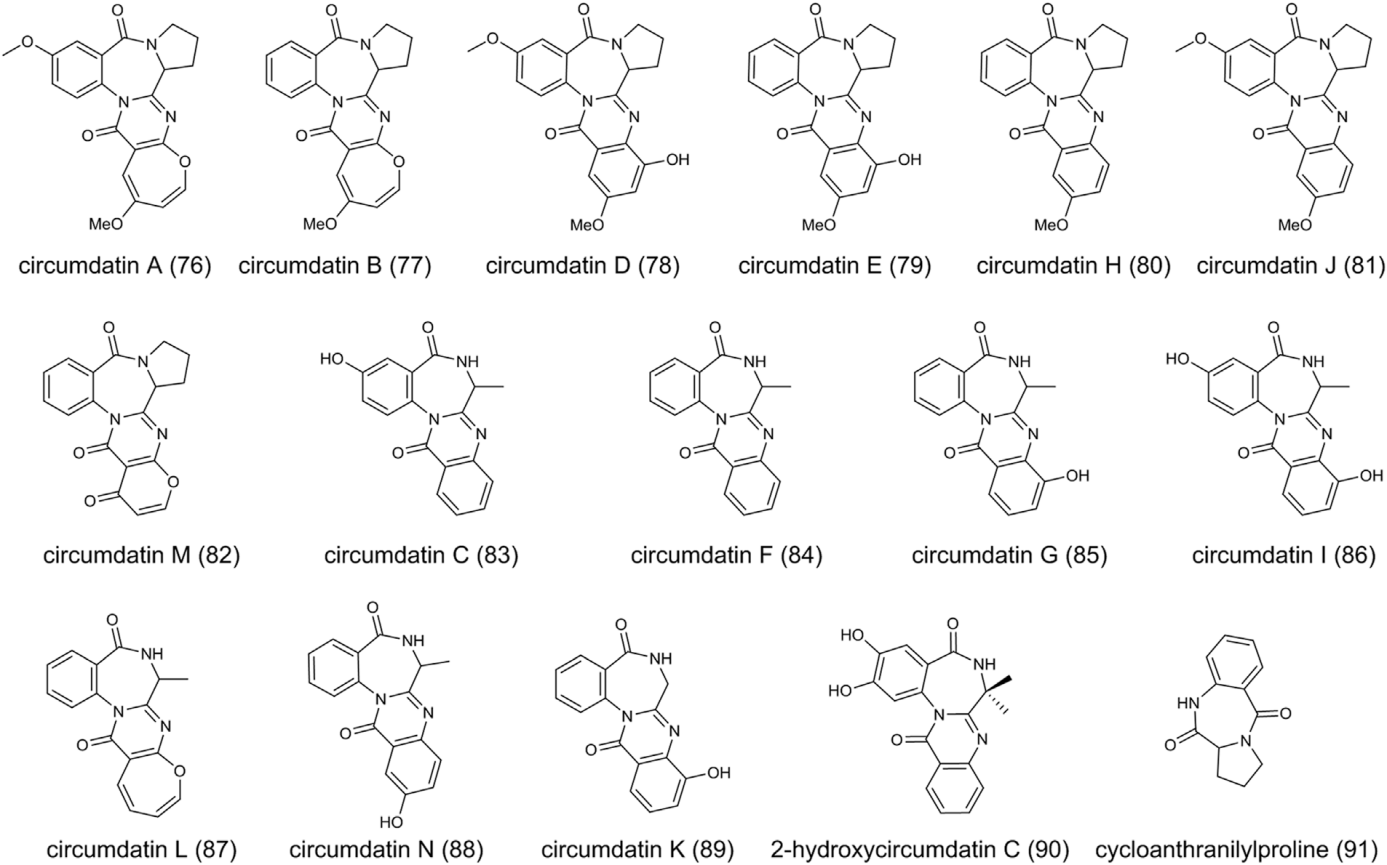
The structure of circumdatins in *Aspergillus ochraceus*.

The circumdatins are a group of benzodiazepine alkaloids, first discovered in 1999 from a terrestrial isolate of the fungus *A. ochraceus* (Rahbæk and Breinholt, 1999). Structurally, the core chemical structure of benzodiazepines is the fusion of a benzene ring and a diazepine ring (Bhathiwal et al., 2022). Until now, thirteen compounds in the circumdatin family have been reported (circumdatins A-N). Biologically, circumdatin derivatives are generated from an amino acid and two anthranilic acids. Structural diversity of circumdatins depends on the type of amino acid and the different substituents. For example, circumdatins A (**76**) (Rahbæk and Breinholt, 1999; Ookura et al., 2008), B (**77**) (Rahbæk and Breinholt, 1999; Ookura et al., 2008), D (**78**) (Rahbæk and Breinholt, 1999), E (**79**) (Rahbæk and Breinholt, 1999), H (**80**) (Lopez-Gresa et al., 2005), J (**81**) (Zhichkin et al., 2010) and M (**82**) (Wang et al., 2019) contain proline, circumdatins C (**83**) (Rahbæk and Breinholt, 1999), F (**84**) (Rahbæk and Breinholt, 1999), G (**85**) (Dai et al., 2001), I (**86**) (Zhang et al., 2008), L (**87**) (Peng et al., 2013) and N (**88**) (Hu et al., 2021) contain alanine and circumdatin K (**89**) (Peng et al., 2013) contains glycine molecular structure, respectively (Figure 6). Most of the compounds were isolated from the genus *Aspergillus*, e.g., *A. ochraceus*, *A. westerdijkiae*, *A. ostianus*, and *A. petrakii*, while the (**86**) was isolated from the genus *Exophiala* (Zhang et al., 2008). In addition, a derivative 2-hydroxycircumdatin C (**90**) has been found in endophytic fungus *A. ochraceus* (Cui et al., 2009). As reported, *A. ochraceus* consistently produces circumdatins. Cycloanthranilylproline (**91**), isolated from *A. ochraceus*, is a kind of benzodiazepine alkaloid while not included in the circumdatin family (Nakatani et al., 2004; Frank et al., 2019). (**91**) derives from a proline and one molecule of anthranilic acid, and contains the fusion of a benzene ring and a diazepine ring.

Circumdatins demonstrated inhibitory activity similar to other inhibitors of the mammalian mitochondrial respiratory chain (Fontana et al., 2001). For example, the IC_50_ value of (**80**) against NADH oxidase is around 1.5 μM, indicating its potential to develop new tools for insect control (Lopez-Gresa et al., 2005). (**83**), (**85**), and (**86**) exhibited an ultraviolet-A protecting activity, which was better than the sunscreen agent oxybenzone (Zhang et al., 2008). (**90**) showed great DPPH radical-scavenging activity, which was more potent than the well-known butylated hydroxytoluene with an IC_50_ value of 9.9 mm (Cui et al., 2009). (**78**) demonstrated potential as an agent for neuroprotective effects by attenuating LPS-induced pro-Inflammatory responses (Zhang et al., 2020). Several compounds have been evaluated for their cytotoxicity, while no evidence provides to prove their cytotoxicity.

The biosynthesis of circumdatin remains to be explored in *A. ochraceus* and other fungi due to its structural complexity. However, the biosynthetic gene cluster of anthramycin and sibiromycin, which belong to the benzodiazepine family, have been identified clearly (Hu et al., 2007; Li et al., 2009), indicating NRPS might be involved in the biosynthesis of circumdatins.

### Pyrazines

**FIGURE 7:**
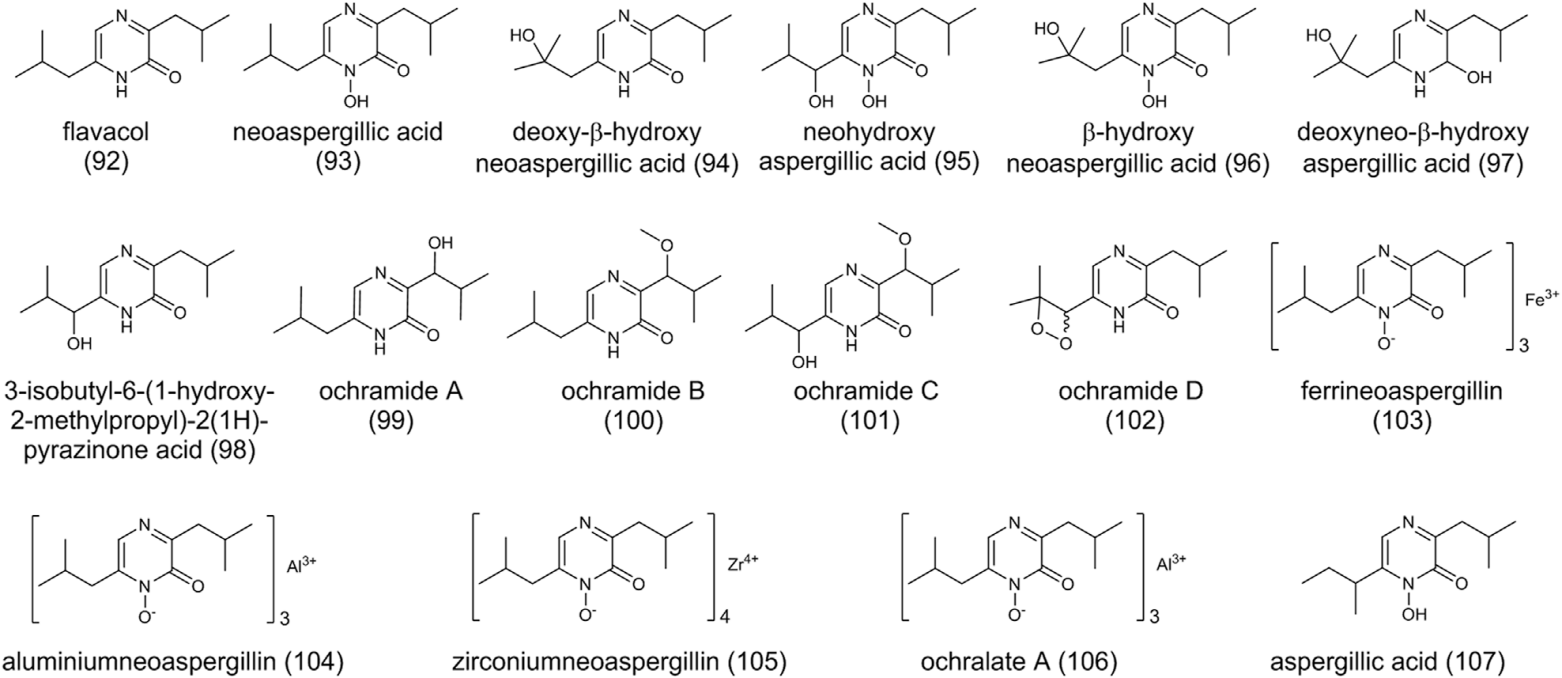
The structure of pyrazines in *Aspergillus ochraceus*.

Pyrazines occur frequently in nature and are produced by plants, animals, and microorganisms (Ong et al., 2017). Several pyrazine compounds have been identified in *A. ochraceus* (Figure 7) and they are screened by bio-activities studies. Flavacol (**92**), neoaspergillic acid (**93**), and deoxy-β-hydroxyneoaspergillic acid (**94**) were first identified as pyrazine metabolites in 1972 (Yamazaki et al., 1972). Subsequently, neohydroxyaspergillic acid (**95**), β-hydroxyneoaspergillic acid (96), deoxyneo-β-hydroxyaspergillic (**97**), and 3-isobutyl-6-(1-hydroxy-2-methylpropyl)-2(1H)-pyrazinone acid (**98**) were also found in *A. ochraceus* (Maebayashi et al., 1978). More recently, ochramides A-D (**99**-**102**) were isolated from the fermentation broth of a marine coral-derived strain in a nutrient-limited medium (Peng et al., 2018). All of these compounds are derived from two leucine molecules with different modifications. It is suggested the first step of modification on the side chain is hydroxylation on the α position, followed by dehydration and rehydration on the β position. Three molecules of neoaspergillin chelate with one atom of iron, one atom of aluminum, or one atom zirconium to form ferrineoaspergillin (**103**), aluminiumneoaspergillin (**104**), and zirconiumneoaspergillin (**105**), respectively. *N*-hydroxy-ochramide B chelating with aluminum forms ochralate A (**106**).

It is reported the pyrazine compounds are biosynthesized from NRPS. The identification of a dihydropyrazine *N*,*N*′-dioxide metabolite proposes a noncanonical NRPS pathway for pyrazine derivatives through genome mining of *Pseudomonas* (Kretsch et al., 2018). A gene cluster containing an NRPS-like encoding gene in *A. flavus* is responsible for the synthesis of aspergillic acid (**107**) by NRPS-like gene inactivation experiment (Lebar et al., 2018). Structurally, (**107**) and (**93**) are closely related isomers. Many fungi from *Aspergillus* spp. can produce (**93**) and its hydroxylated analogs, and the genome mining shows they all harbor the homologs BGC of (**107**), indicating the same biosynthesis pathway between (**107**) and (**93**) (Lebar et al., 2019).

### Bis-Indolyl Benzenoids

**FIGURE 8:**
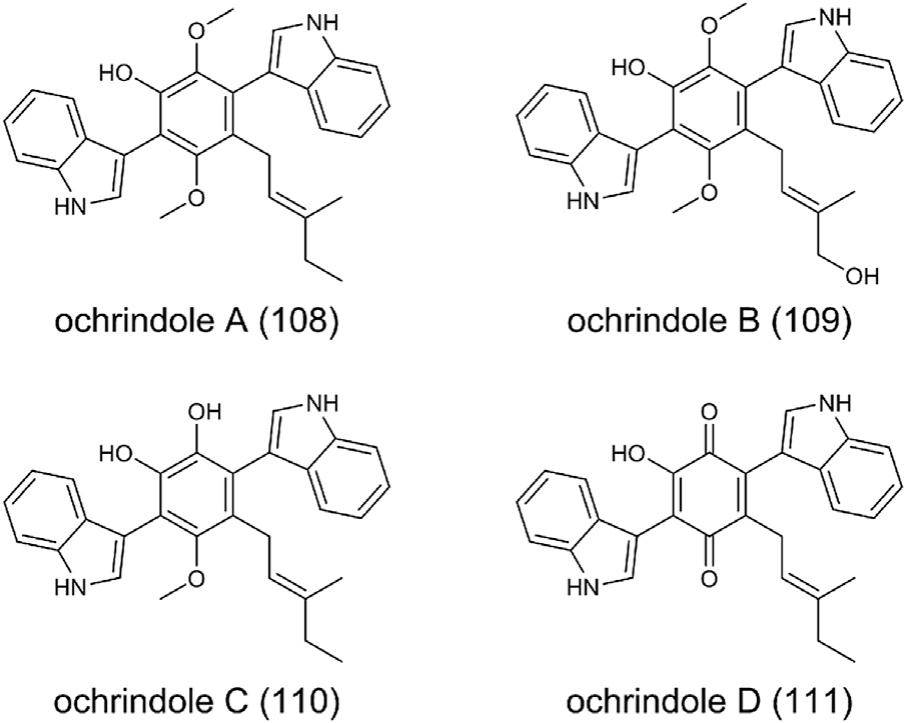
The structure of bis-indolyl benzenoids in *Aspergillus ochraceus*.

Ochrindoles A-D (**108**-**111**) were isolated from the sclerotia of *A. ochraceus* (de Guzman et al., 1994) (Figure 8). Structurally, (**108**-**110**) are bis-indolyl benzenoids, and (**111**) is bis-indolyl quinone. Members of these bis-indolyl structures have been reported from fungal metabolites, such as terriquinones from *A. terreus* (Balibar et al., 2007), kumbicins from *A. kumbius* (Lacey et al., 2016), asterriquinol from *A. sclerotiorum* (Whyte et al., 2000). These compounds typically contain prenyl groups at various positions on the indole moieties or the central benzenoid ring. The biosynthesis of bis-indolyl benzenoids and quinone has been extensively investigated. Briefly, an aminotransferase converts *l* -tryptophan to the indole pyruvic acid, and two molecules of indole pyruvic acid are dimerized by a single-module NRPS to form the bis-indolyl benzenoid skeleton. Then, the oxidoreductase, prenyltransferase, methytransferase successively play the catalytic function to form the corresponding products. Ochrindoles showed moderate activity against the corn earworm *Helicoverpa zea*, fungivorous beetle *Carpophilus bemipterus*, as well as bacterial *Bacillus subtilis* (de Guzman et al., 1994).

### Nitrobenzoyl Sesquiterpenoids

**FIGURE 9:**
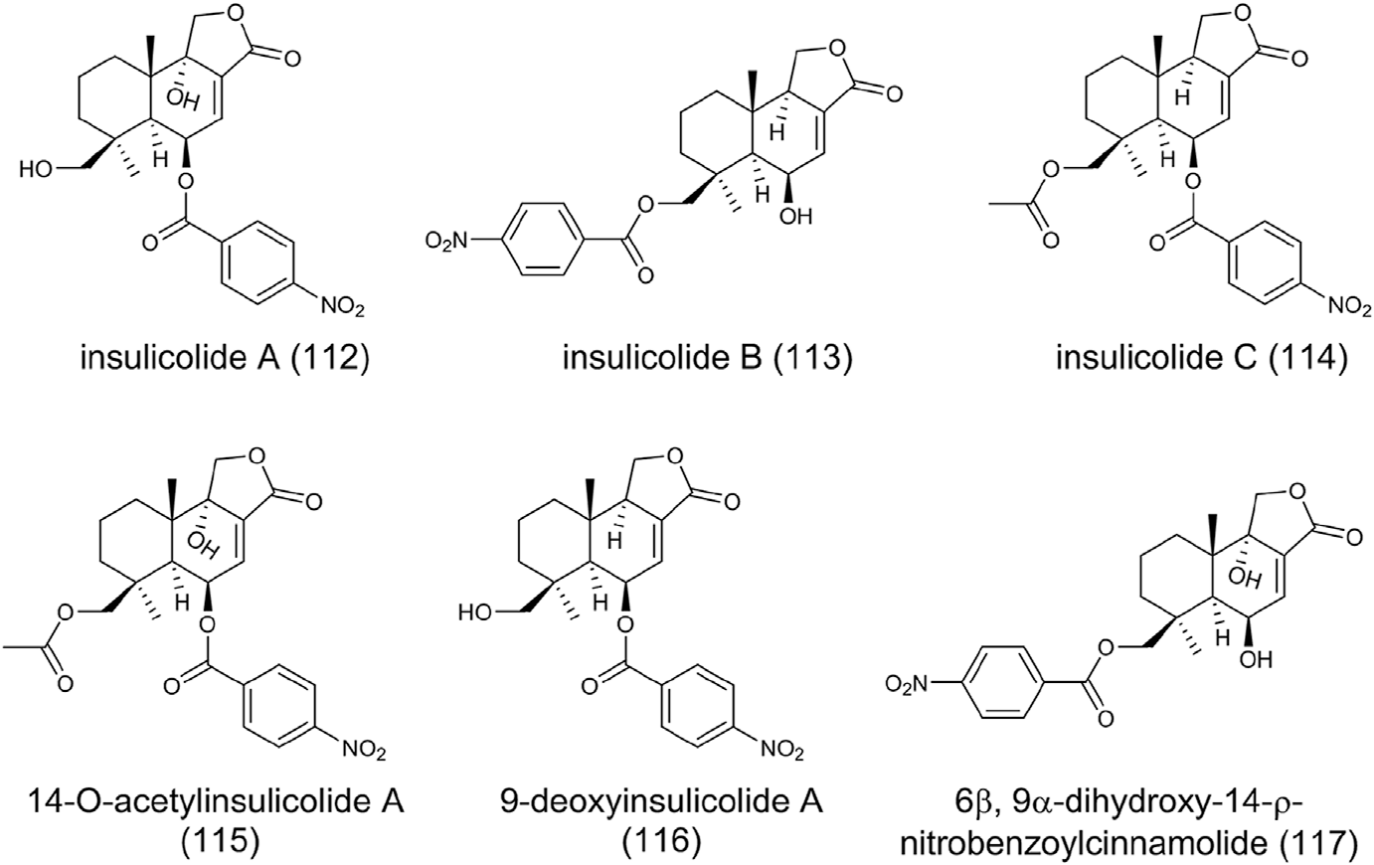
The structure of nitrobenzoyl sesquiterpenoids in *Aspergillus ochraceus*.

Sesquiterpenoids are abundant in nature, while nitrobenzoyl sesquiterpenoids are rare from natural sources. Until now, only several nitrobenzoyl sesquiterpenoids have been identified from marine-derived fungi *A. ochraceus* and *A. insulicola* (Belofsky et al., 1998; Fang et al., 2014; Tan et al., 2018). Insulicolides A-C (**112**-**114**), 14-O-acetylinsulicolide A (**115**), 9-deoxyinsulicolide A (**116**), and 6β, 9α-dihydroxy-14-ρ-nitrobenzoylcinnamolide (**117**) were isolated and characterized from marine-derived *A. ochraceus* (Figure 9)*.* The significant inhibitory activities against the growth of renal carcinoma cells indicate these compounds possess antitumor potential (Tan et al., 2018).

### Steroids

**FIGURE 10:**
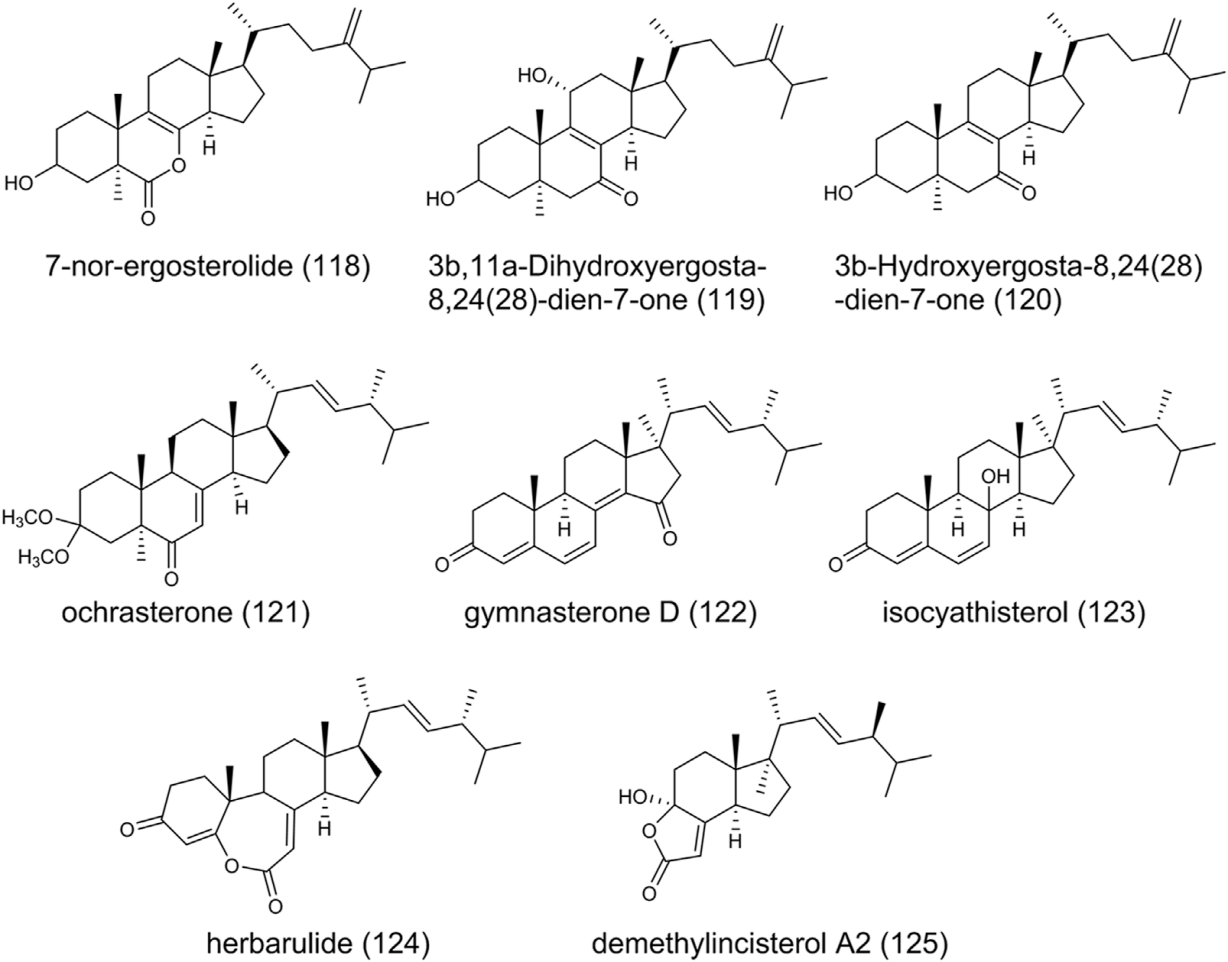
The structure of steroids *Aspergillus ochraceus*.

Steroids function as components of cell membranes or signaling molecules in living cells, with four rings arranged in a specific molecular configuration. As shown in Figure 10, 7-nor-ergosterolide (**118**), featured by a γ, δ-unsaturated pentalactone B-ring system, is the first 7-norsteroid of naturally occurring and isolated from *A. ochraceus*. In addition, 3β,11α-Dihydroxyergosta-8,24(28)-dien-7-one (**119**) and 3β-Hydroxyergosta-8,24(28)-dien-7-one (**120**) were identified from the same fungal strain and exhibited selective cytotoxic activity against tumor cell lines (Cui et al., 2010). Recently, a new ergostane-type sterol derivative ochrasterone (**121**), gymnasterone D (**122**), isocyathisterol (**123**), herbarulide (**124**), and demethylincisterol A2 (**125**) have been obtained (Hu et al., 2021; Tong et al., 2022). (**123**) was first discovered from *A. ustus* with weak antibacterial activity (Liu et al., 2014). Previously, in a bioactivity-guided search for new compounds in a marine sponge *Homaxinella* sp., the degraded (**125**) displayed significant cytotoxicity when it was tested against a panel of five human solid tumor cell lines (Mansoor et al., 2005).

### Others

**FIGURE 11:**
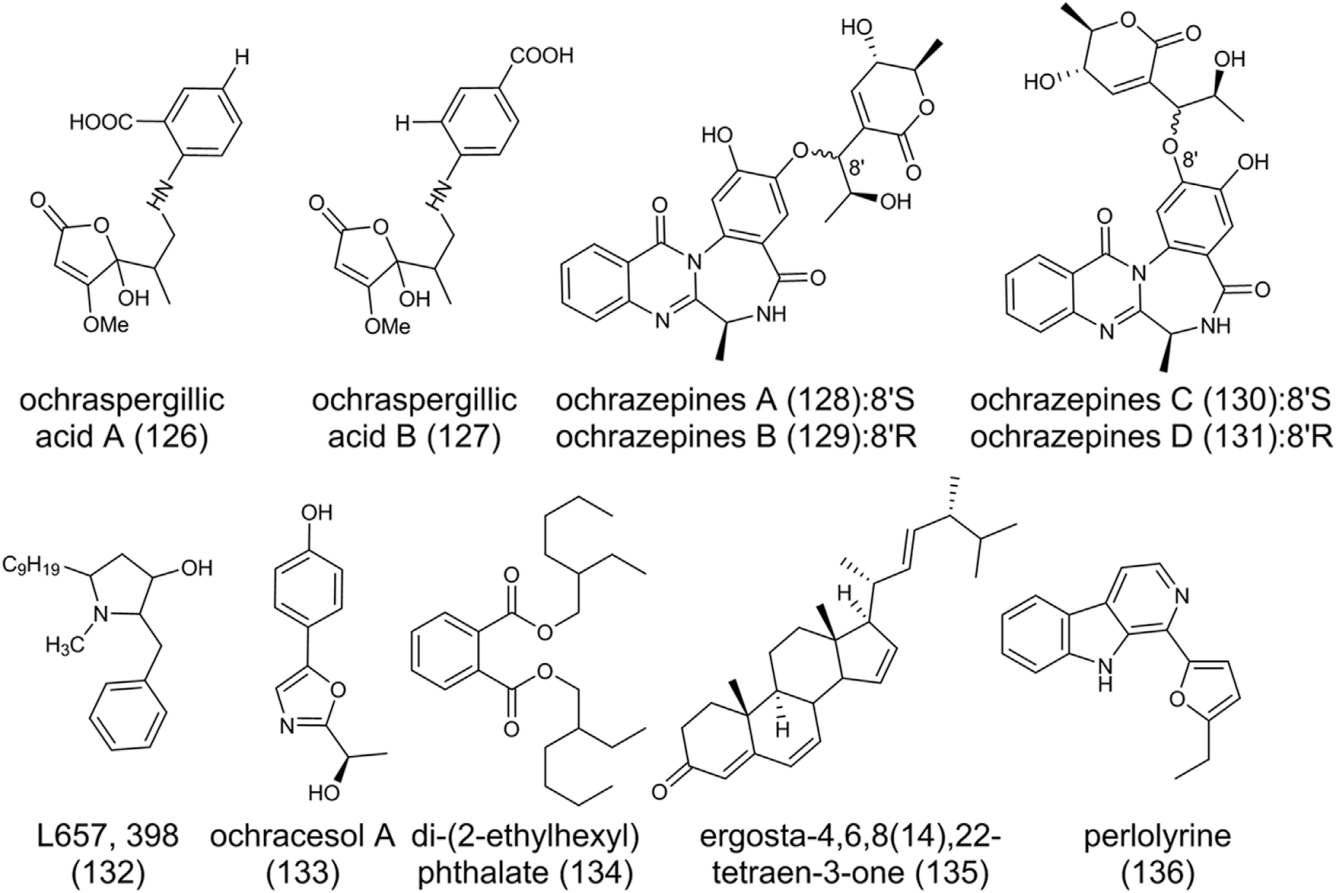
The structure of other secondary metabolites in *Aspergillus ochraceus*.

As shown in Figure 11, alkaloids ochraspergillic acids A (**126**), B (**127**), the adducts of dihydropenicillic acid (**31**) and ο- or ρ-aminobenzoic acid, were produced when *A. ochraceus* co-culture with *Bacillus subtilis* (Frank et al., 2019). Feeding experiments by adding either anthranilic acid or *l* -tryptophan to a solid rice medium also demonstrated the production of (**126**), indicating anthranilic acid and *l* -tryptophan are building blocks of ochraspergillic acids. Ochrazepines A-D (**128**-**131**) are dimerized from 2-hydroxycircumdatin C **(90)** and aspyrone (**10**) through a nucleophilic addition to epoxide (Fan et al., 2019). Semi-synthesis by nucleophilic addition reactions confirmed the speculation that (**10**) possibly underwent a S_N_1-like process to form the more stable allyl carbon positive ion, immediately followed by reaction with the oxygen anions of **(90)** to yield two pairs of epimers (**128)**/(**129)** and (**130)**/(**131)**, respectively. The change of bioactivity of these compounds due to conjugation indicated the formation of hybrids provides more natural products for bioactivity studies. L657,398 (**132**), with broad antifungal activity, is a pyrollidine isolated from the mycelium of *A. ochraceus* in liquid fermentation (Schwartz et al., 1988). Ochracesol A (**133**), which contains an oxazole ring, exhibited anti-PD activities on SH-SY5Y cells (Hu et al., 2021). Di-(2-ethylhexyl) phthalate (**134**), ergosta-4,6,8 (14),22-tetraen-3-one (**135**), and a beta-carboline alkaloid perlolyrine (**136**) have also been found in *A. ochraceus* (Hu et al., 2021).

## Discussion

Common fungi, especially *A. ochraceus*, are regularly underestimated for their biosynthetic potential, which deserves our recognition. Here, we comprehensively review the known SMs produced by *A. ochraceus*, and discuss their bioactivities and biosynthetic pathway. *A. ochraceus* produces a range of polyketides, nonribosomal peptides, diketopiperazine, terpenes, and other alkaloids. Except for the mycotoxins OTA, (**6**) and (**7**), these compounds possess antimicrobial, antiviral, anti-insect, antitumor, antioxidant, and anti-inflammatory activities. Thus, *A. ochraceus* strains could be valuable sources of compounds in the areas of medicine and agriculture.

In terms of fungi, a large number of natural products have been isolated from *A. ochraceus* until now. Nonetheless, several strategies have been used for enhancing the chemical diversity of microorganisms. Different media used in the cultivation of *A. ochraceus* leads to the production of different compounds. For example, when several inorganic salts or organic supplements are added to the solid rice medium culture, *A. ochraceus* is found to produce different metabolites, resulting in discovering the novel compounds; and this strategy verifies the OSMAC (One Strain MAny Compounds) theory (Frank et al., 2019). Grimm-Allen iron-limited medium allows *A. ochraceus* to secrete the extracellular siderophores (Jalal et al., 1984). Furthermore, the cultivation of two different microbial strains (*A. ochraceus* and *B. subtilis*) together leads to the induction of (**126**) and (**127**), which are not previously observed in the independent culture of each strain (Frank et al., 2019). Genetically, the changes in the cultural environment alter the gene expression profiles, hence activating silent SM gene clusters (Keller, 2019). For example, a total of 64 backbone SM genes, which are responsible for the biosynthesis of the chemical skeleton, are differentially expressed when *A. nidulans* undergoes a fungal-fungal cocultivation, leading to the activation of 14 aspernidine derivatives (Wang et al., 2022b).

Although a large number of compounds have been discovered in *A. ochraceus*, some of them are derived from the same biosynthetic pathway, e.g., (**8**) and (**9**) (Harris and Mantle, 2001). In fact, the number of genes encoding biosynthetic enzymes clearly outnumbers the identified compounds in *A. ochraceus* (Gonçalves et al., 2021). Finally, we remind the scientific community not to discredit the ability of *A. ochraceus* to produce natural products and encourage them to explore unexpected natural products through manipulating nutritional or environmental factors.
